# Evaluation of the diabetes in pregnancy study group of India criteria and Carpenter-Coustan criteria in the diagnosis of gestational diabetes mellitus

**DOI:** 10.4274/tjod.57255

**Published:** 2018-06-21

**Authors:** Shazia Khan, Himadri Bal, Inam Danish Khan, Debashish Paul

**Affiliations:** 1 INHS Kalyani Hospital, Clinic of Obstetrics and Gynecology, Visakhapatnam, India; 2 Dr. DY. Patil Medical College Hospital and Research Centre, Clinic of Obstetrics and Gynecology, Pune, India; 3 Army College of Medical Sciences and Base Hospital, Clinic of Microbiology, New Delhi, India; 4 Armed Forces Medical College, Clinic of Obstetrics and Gynecology, Pune, India

**Keywords:** Gestational diabetes mellitus, Diabetes in Pregnancy Study Group of India criteria, Carpenter Coustan criteria, pregnancy, glucose tolerance test

## Abstract

**Objective:**

Gestational diabetes mellitus (GDM) is defined as any degree of glucose intolerance that is diagnosed for the first time during pregnancy. This prospective study was undertaken to validate the single-step non-fasting 75 gm Diabetes in Pregnancy Study Group of India (DIPSI) criteria of GDM in Indian patients in comparison with the two-step fasting 100 gm glucose challenge through the Carpenter Coustan criteria (CCC).

**Materials and Methods:**

Two hundred patients underwent comparative testing using the DIPSI criteria and CCC. Plasma venous blood glucose levels were estimated using the hexokinase method; values ≥140 mg/dL at 2 hours were considered positive according to the DIPSI criteria. Any two values from ≥95 mg/dL for fasting, ≥180 mg/dL at 1 hour, ≥155 mg/dL at 2 hours, and ≥140 mg/dL at 3 hours were considered positive with the CCC.

**Results:**

The mean age and body mass index were 24.26±3.75 years and 20.7±3.07 kg/m2. The sensitivity, specificity, and positive and negative predictive values of the DIPSI guidelines were found as 100%, 97.14%, 83.87%, and 100%, respectively. The positive and negative likelihood ratios were 35.8 and zero. Diagnostic accuracy was found as 97.56%.

**Conclusion:**

DIPSI having high sensitivity, specificity, negative predictive value and diagnostic accuracy. DIPSI offers simplicity, feasibility, convenience, and repeatability while economizing universal screening and diagnosis of GDM on a mass-scale. The DIPSI procedure has the potential to be applied to the entire obstetric population, in the implementation of public health programs to diagnose GDM in the community, thus reaching the needs of the developing world.

**PRECIS:** Diabetes in pregnancy study group of India criteria versus carpenter coustan criteria for diagnosing gestational diabetes mellitus.

## Introduction

Gestational diabetes mellitus (GDM) is defined as any degree of glucose intolerance that is diagnosed for the first time during pregnancy, irrespective of treatment with diet or insulin^(1,2)^. GDM predisposes to future risk of type-2 DM in both the mother and her offspring^(3)^. Twenty to fifty percent of women with GDM will develop type-2 DM in the 5-10 years after delivery, corresponding to a 7.4-fold increased risk. Untreated GDM during pregnancy may lead to an increased risk of large-for-gestational-age births, low blood sugar, and jaundice in the neonatal period^(4,5)^. The prevalence of GDM is increasing worldwide proportionate to DM in the population. GDM occurs in up to 14% of all pregnancies in the United States of America (USA), whereas Asians have an 11.3 higher relative-risk of GDM^(6)^. Indian women with GDM have a higher risk of diabetes and metabolic syndrome^(7)^. Early detection of glucose intolerance during pregnancy has tri-pronged implications. One, GDM offers a timely opportunity for screening, management, and prevention of GDM and type-2 DM in pregnant women. Secondly, it prevents fetal complications thereby improving neonatal outcomes^(8)^. Thirdly, it offers the development, testing, and implementation of clinical and epidemiologic strategies for diabetes prevention in the population^(9)^. In the absence of consensus-based guidelines for the screening and diagnosis of GDM, there are variations in antenatal-care protocols^(10)^. There are variations between the American Diabetes Association (ADA) recommendations of selective screening vis-a-vis the American College of Obstetricians and Gynecologists (ACOG), which recommends universal screening. Universal mandatory screening for GDM is becoming the standard of antenatal-care even in low-income countries, notwithstanding healthcare equity and accessibility. Most institutions offer a 2-step procedure for screening and diagnosis of GDM as per the Carpenter Coustan criteria (CCC), which is cumbersome and entails additional costs to the exchequer. The international hyperglycemia and pregnancy outcome study results were promulgated by the International Association of Diabetes and Pregnancy Study Groups (IADPSG), which recommended a single-step testing methodology, reducing costs, and improving patient convenience. The IADPSG thresholds of fasting >92 mg/dL, 1 hour ≥180 mg/dL, or 2 hour ≥153 mg/dL plasma venous glucose values after a 75 g oral glucose tolerance test (OGTT) were accepted by the World Health Organization (WHO) and the ADA in 2013 and 2014, respectively, despite having been reported as having lower sensitivity^(11,12,13)^. The Diabetes in Pregnancy Study Group of India (DIPSI) criteria are a major breakthrough because they cater for the screening and diagnosis of all pregnant women irrespective of fasting state through a simple, economical, and convenient single-step procedure with a 75 g 2 hour glucose test with a cut-off point of >140 mg/dL for diagnosis. This prospective study was undertaken to validate the single-step non-fasting 75 g DIPSI criteria of GDM in Indian patients in comparison with the two-step fasting 100 g OGTT with the CCC^(14)^.

## Materials and Methods

The prospective comparative triple-blind study was conducted with 200 pregnant women who presented to the antenatal clinic of a 1600-bed tertiary-care teaching hospital in Western India over a period of two years from May 2012 to April 2014, after obtaining written informed consents and approval from Armed forces Medical Coleges Ethics Committee. All pregnant women with recorded ≤20 weeks period of gestation (POG) were included. Patients with a history of GDM/impaired glucose tolerance/DM, unexplained stillbirth, a macrosomic baby, congenital anomalies or birth injuries were excluded. Triple-blinding of patients, gynecologists, and pathologists was ensured to eliminate confounding and bias. All 200 patients were subjected to comparative testing through a non-fasting 75 g oral glucose (DIPSI) and fasting 100 g OGTT interpreted by CCC at less than 20 weeks POG and again between 24-28 weeks POG, with a temporal separation of ≤4 days between the non-fasting OGTT with the DIPSI criteria and fasting OGTT with CCC. Plasma venous blood glucose levels were estimated using the hexokinase method on an autoanalyzer (Siemens Healthcare Diagnostics, Inc., West Sacramento, CA 95691 USA). Values ≥140 mg/dL at 2 hours were considered positive with the DIPSI criteria. Any two values from ≥95 mg/dL for fasting, ≥180 mg/dL at 1 hour, ≥155 mg/dL at 2 hours, and ≥140 mg/dL at 3 hours were considered positive with the 100 g OGTT with the CCC for the diagnosis of GDM. Quality control was ensured using internal quality control kits, Levey-Jennings charts based on lab-derived mean and standard deviation, corrective action on violations of Westgard rules, and subscribed external quality controls.

### Statistical Analysis

Data were analyzed using SPSS (version 21; IBM Corporation). The patients’ clinicodemographic profiles and blood glucose levels were correlated for descriptive statistics including frequency, percentages, and 95% confidence intervals (CI).

## Results

The study had a 100% follow-up with no drop-outs. The mean age and body mass index (BMI) of the patients were 24.26±3.75 years and 20.7±3.07 kg/m^2^. Of the 200 women, 31/200 (15.5%, 95% CI: 10.93-21.44) tested positive with the DIPSI criteria, and 26/200 (10.5%, 95% CI: 6.77-15.81) tested positive in the 100 gm OGTT as per the CCC. The 169 women who initially tested negative with the DIPSI criteria continued to be negative on repeat testing with the DIPSI and CCC at 24-28 weeks POG. The prevalence of GDM in the study cohort was found as 15.5% using DIPSI criteria, and the prevalence of GDM after 100 gm OGTT with the CCC was 13% (Table 1). The sensitivity, specificity, and positive and negative predictive values of the DIPSI guidelines were found as 100%, 97.14%, 83.87%, and 100%, respectively. The positive and negative likelihood ratios were 35.8 and zero. Diagnostic accuracy was found as 97.56% (Table 2).

## Discussion

The effectiveness of glucose-challenge tests in the non-fasting state for screening and diagnosing GDM has long been a matter of debate. The ADA recommends only selective screening for GDM. Selective screening by risk factors such as woman’s age, ethnicity, and BMI may miss some patients with GDM in the lower risk category, whereas more such patients may be diagnosed in the higher risk category. The reason for universal screening for GDM is to try and reduce the number of pregnant women undergoing OGTTs. A universal screening protocol requires the consideration of patient comfort, cost, and the risk of missing the diagnosis. The current ACOG recommendation of universal screening is a more practical approach but it advocates universal screening using two-step methods. Currently, the most used screening test is the oral glucose challenge test (OGCT) with 50 g of glucose followed by an OGTT with 100 g of glucose. Other screening tests and cut-off values are fasting blood glucose (126 mg/dL, 7.0 mmol/L) and random blood glucose (200 mg/dL, 11.1 mmol/L). The diagnostic test for GDM has always been the 100 g 3 hour OGTT. The WHO-IADPSG 75 g OGTT is currently recommended by the WHO for the diagnosis of GDM and it is widely used in Europe. The 100-gm OGTT is still predominantly used in the USA. However, in countries such as Saudi Arabia, Nigeria, and China, a 1 hour 50 g OGCT at 24-28 weeks of gestation is considered as a reliable universal screening test for GDM. Measurements of blood glucose levels in capillary bloods using a glucometer has made screening easy and simple because it can be performed in an office setting and does not require elaborate laboratory facilities, which may be far and few in resource-limited healthcare environments. It is important to know that capillary blood glucose levels are comparable to venous blood glucose levels during the fasting state but are higher after meals^(15)^. Most institutions offer a 2-step procedure for screening and diagnosis of GDM, under ADA, ACOG, WHO-IADPSG, Canadian Diabetes Association, National Diabetes Data Group (NDDG), National Institute of Health and Care Excellence in the United Kingdom and/or Australasian criteria. Reproducibility has been reported as 78% at best. In the 4^th^ International Workshop Conference on GDM in 1997, a consensus was reached on replacing NDDG criteria with CCC criteria, which have lower threshold values for the diagnosis of GDM^(16,17,18)^. The screening and diagnosis of GDM has been simplified from the two-step fasting OGTT under ADA criteria/CCC to single-step fasting OGTT under WHO-IADPSG criteria. The 75 g DIPSI criteria with a 2 hour cut-off value of ≥140 mg/dL is a notch simpler than the WHO-IADPSG criteria because it offers both screening and diagnosis with a single-step non-fasting OGTT, which is immensely practical, economical, feasible, and convenient for patients and obstetric healthcare providers. The DIPSI criteria offer a promising technique with a high sensitivity of 100%, specificity of 97.21%, accuracy of 97.56%, and negative predictive value of 100%, compared with the  fasting 100 g OGTT as per the CCC as seen in this study. Various studies have shown higher sensitivity and specificity of non-fasting 75 g two hour DIPSI testing compared with other criteria^(19)^. The DIPSI criteria have demonstrated 100% sensitivity, 100% specificity, and 94% diagnostic accuracy^(20,21,22,23)^.  Non-fasting OGTT causes the least disturbance to a pregnant woman’s routine activities. Even if the DIPSI test is to be repeated in each trimester, the cost of performing DIPSI procedures will be less than the cost of performing any other diagnostic procedures because it requires little preparation, without requiring the prior interposition of the screening test. DIPSI has been proven to be a suitable test with higher sensitivity than WHO-IADPSG criteria in consonance with this study^(24,25)^. The DIPSI criteria have limitations in comparison with other OGTT criteria as seen in different patient populations, which may be a doubled-edged decision conundrum. The DIPSI criteria may not be able to account for fasting hyperglycemia. False-positive GDM with the DIPSI criteria in the absence of confirmatory GDM tested by other OGTT with low PPV, can lead to psychological stress, clinico-ultrasonographic surveillance, and interventions. False-negative GDM with DIPSI may be labeled as normal and may impact fetomaternal outcomes. DIPSI is based on the observations of the diabetes in pregnancy and awareness project. OGTT irrespective of last meal timing to diagnose GDM has been proven, which is in accordance to DIPSI guidelines. It is important to accept that no test is 100% sensitive or specific or has a 100% PPV and NPV. The WHO-IADPSG criteria have been reported to have lower sensitivity. Certain studies reported a lower sensitivity of DIPSI in comparison with the 75 g OGTT; however, almost all of these studies also reported high specificity and negative predictive values of DIPSI^(26,27,28)^. The high negative predictive value with a 75 g non-fasting DIPSI can definitely rule out GDM, thus making DIPSI a convenient and cost-effective screening tool for outpatients in antenatal centers^(29,30,31)^. However, the approach has limitations and cannot be concluded as superior to the universal approach with this study. Indian studies reported the prevalence of GDM as between 16.55% and 22% using the DIPSI criteria, which is comparable to the prevalence of 15.5% in this study^(32,33)^. Challenges in laboratory quality control exist in developing countries conducting mass-screening in resource limited facilities, which affects clinical decision- making^(34)^. The DIPSI criteria have been included in the guidelines of the Ministry of Health and Family Welfare, Government of India^(35,36,37)^.

### Study Limitations

The study is limited by sample size and unaccounted fasting hyperglycemia.

## Conclusion

The DIPSI criteria have high sensitivity, specificity, negative predictive values and diagnostic accuracy. DIPSI offers simplicity, feasibility, convenience, and repeatability, while economizing universal screening and diagnoses of GDM on a mass-scale. The DIPSI procedures have the potential to be applied to the entire obstetric population, in the implementation of public health programs to diagnose GDM in the community, thus reaching the needs of the developing world.

## Figures and Tables

**Table 1:**
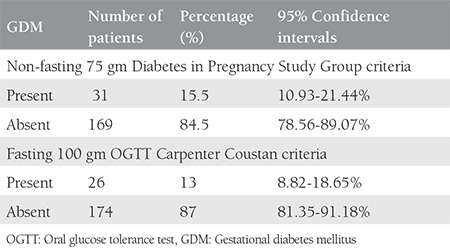
Prevalence of gestational diabetes mellitus (n=200)

**Table 2:**
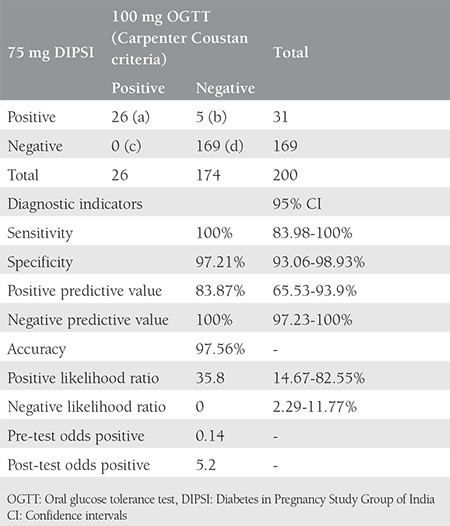
Evaluation of non-fasting 75 gm Diabetes in Pregnancy Study group criteria (n=200)
